# Preparation, Supramolecular Aggregation and Immunological Activity of the Bona Fide Vaccine Adjuvant Sulfavant S

**DOI:** 10.3390/md18090451

**Published:** 2020-08-29

**Authors:** Emiliano Manzo, Laura Fioretto, Carmela Gallo, Marcello Ziaco, Genoveffa Nuzzo, Giuliana D’Ippolito, Assunta Borzacchiello, Antonio Fabozzi, Raffaele De Palma, Angelo Fontana

**Affiliations:** 1 Bio-Organic Chemistry Unit, CNR-Institute of Biomolecular Chemistry, Via Campi Flegrei 34, 80078 Pozzuoli, Italy; l.fioretto@icb.cnr.it (L.F.); carmen.gallo@icb.cnr.it (C.G.); nuzzo.genoveffa@icb.cnr.it (G.N.); gdippolito@icb.cnr.it (G.D.); raffaele.depalma@unige.it (R.D.P.); 2 Consorzio Italbiotec, Via Fantoli, 16/15, 20138 Milano, Italy; 3 BioSearch Srl., Villa Comunale c/o Stazione Zoologica “A.Dohrn”, 80121 Napoli, Italy; m.ziaco@icb.cnr.it; 4 Institute for Polymers, Composites and Biomaterials (IPCB), CNR, 80125 Naples, Italy; bassunta@unina.it (A.B.); Sirfabozzi@hotmail.it (A.F.); 5 Medicina Interna, Immunologia Clinica e Medicina Traslazionale, Università di Genova and IRCCS-Ospedale S. Martino, 16131 Genova, Italy

**Keywords:** sulfavants, adjuvant, immunomodulatory activity, colloid, aggregates

## Abstract

In aqueous conditions, amphiphilic bioactive molecules are able to form self-assembled colloidal structures modifying their biological activity. This behavior is generally neglected in preclinical studies, despite its impact on pharmacological development. In this regard, a significative example is represented by a new class of amphiphilic marine-inspired vaccine adjuvants, collectively named Sulfavants, based on the β-sulfoquinovosyl-diacylglyceride skeleton. The family includes the lead product Sulfavant A (**1**) and two epimers, Sulfavant R (**2**) and Sulfavant S (**3**), differing only for the stereochemistry at C-2 of glycerol. The three compounds showed a significant difference in immunological potency, presumably correlated with change of the aggregates in water. Here, a new synthesis of diastereopure **3** was achieved, and the study of the immunomodulatory behavior of mixtures of **2/3** proved that the bizarre in vitro response to **1**–**3** effectively depends on the supramolecular aggregation states, likely affecting the bioavailability of agonists that can effectively interact with the cellular targets. The evidence obtained with the mixture of pure Sulfavant R (**2**) and Sulfavant S (**3**) proves, for the first time, that supramolecular organization of a mixture of active epimers in aqueous solution can bias evaluation of their biological and pharmacological potential.

## 1. Introduction

Development of new drugs is a long and expensive process, and often many unpredictable problems arise in clinical phases due to the chemical-physical behavior of some molecules in physiological environments. Several pharmacologically active compounds and commercial drugs are in fact amphiphilic substances able to self-aggregate in aqueous solutions. Self-assembly in water is a spontaneous process involving the arrangement in supramolecular structures that are stabilized by non-covalent interactions and minimizes the direct contact between the hydrophobic part of the molecule and the polar solvent [1]. This behavior is as common as it is poorly considered, even if it can seriously affect biological activity and pharmacological developments. Only recently, studies have focused on the relevance of physicochemical properties, such as lipophilicity, in the in vitro selection of drug candidates and likelihood of success in development [2]. In fact, molecular aggregation can be critical in determining in vivo ADMET (absorption, distribution, metabolism, excretion, and toxicity) properties, but also in affecting the overall quality of a drug candidate in cellular assays. In physiological media, lipophilic substances produce complex equilibria involving free molecules and many aggregates differing in size and shape. Therefore, as aggregates are not involved in the pharmacodynamic interaction, these equilibria determine the effective concentration of the bioactive product on the target and in cellular tests.

Adjuvants are chemical components that are combined with antigens to enhance the immune response to vaccines [3]. Traditionally, adjuvants are composed of a suspension of insoluble compounds (e.g., oils, aluminum, particulate materials containing small molecules) in water. In the last few years, a major breakthrough has been the discovery of the link between adjuvants and innate immune response triggered by antigen-presenting cells (APCs) that capture and process antigens for presentation to T-lymphocytes, and to produce signals required for the proliferation and differentiation of lymphocytes [4,5,6,7,8,9,10,11]. In particular, the identification of pattern recognition receptors (PRRs) as primary effectors of the plastic activation of APCs has rapidly led to the rational design of molecular adjuvants based on single, immunomodulatory molecules. In this context, we recently characterized β-sulfoquinovosyl-diacyl glycerols (β-SQDGs) as a novel class of vaccine adjuvants collectively named Sulfavants. These synthetic molecules were inspired by natural and marine α-sulfoquinovosyl-diacylglycerols (α-SQDGs) occurring as membrane constituents in photosynthetic organisms [12,13,14,15]. Sulfavant A (**1**) (1,2-*O*-distearoyl-3-*O*-(β-sulfoquinovosyl)-*R/S*-glycerol), the prototype of the family, induces maturation of human dendritic cells (hDCs) at micromolar concentrations with a typical “bell-shaped” dose–response curve that is featured by a maximum around 10 μM. Sulfavant A (**1**) also showed promising adjuvant activity in *in vivo* experiments, as it was able both to boost immune protection in mice and to inhibit tumor growth in an experimental model of cancer vaccine against melanoma [13,14,15]. 


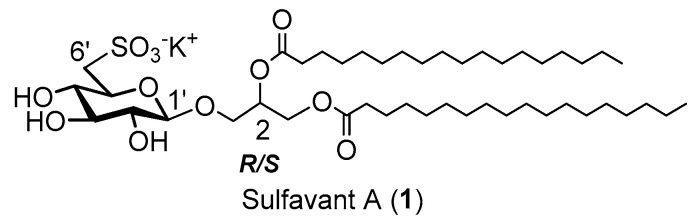


**Scheme 1:**
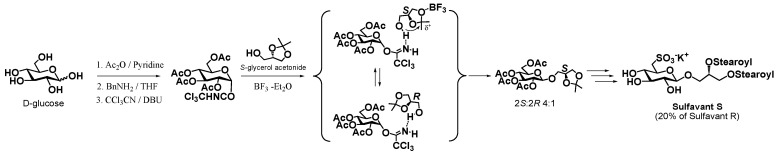
Literature synthetic approach for the preparation of Sulfavant S (**3**) (with 20% of *R* epimer) according to Manzo et al., 2019 [12].

Compound **1** is a 1.3:1 mixture of *R/S* epimers at carbon 2 of glycerol moiety. In order to investigate the pharmacological properties of this new class of molecules, we also synthetized two enantiopure analogues named Sulfavant R (**2**) and S (**3**) [12]. Surprisingly, compound **2** showed maturation of hDCs at nanomolar concentrations with 1000-fold increase of the activity in comparison to the epimeric mixture **1**. We never had the opportunity to test rigorously the biological response to pure Sulfavant S (**3**) because of a partial loss of stereospecificity of the synthesis for this product due to a fast process of opening and closure of the *S*-glycerol acetonide during the glycosylation step (Scheme 1) [12]. However, a fraction containing 80% Sulfavant S (**3**) and 20% Sulfavant R (**2**) was also active on DCs but at concentrations between those of **1** and **2**. All of these compounds gave bell-shaped concentration–response curves, and chemo-physical analysis revealed a clear correlation between size of the microparticles in water and activation of hDC maturation [12]. 
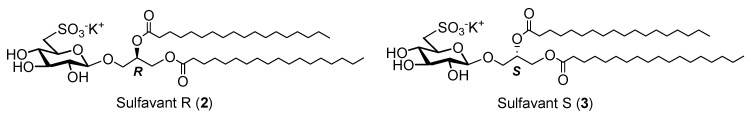


In the present work, we implemented a stereospecific synthesis of Sulfavant S (**3**) with the aim to prove that the immunological priming of hDC by the enantiopure isomers is significantly dependent, per se, on the type of aggregation of active products. 

## 2. Results and Discussion

**Scheme 2:**
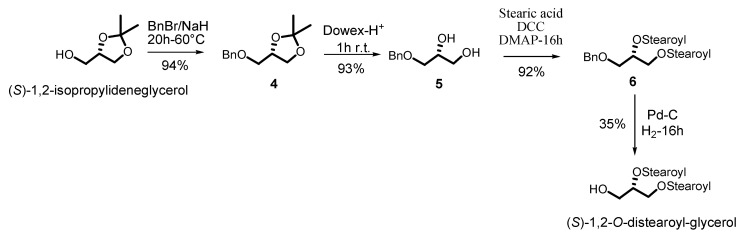
Synthesis of the acceptor (*S*)-1,2-*O*-distearoylglycerol.

The use of (*R*)-(-)-1,2-isopropylideneglycerol as an acceptor in the coupling step with the trichloroacetamidate sugar gave high stereospecificity for the synthesis of Sulfavant R (**2**) [12]. However, a similar reaction with (*S*)-(+)-1,2-isopropylideneglycerol led to a low diastereoselective yield because of opening and closure of the acetonide ring under acidic conditions due to the boron trifluoride catalyst. As described in Scheme 1, formation of a complex between boron trifluoride and the acetonide oxygen atoms can start a fast and reversible process of racemization at C-2 of glycerol. The rearrangement rate was comparable to formation of the glycosidic bond, thus resulting in 20% epimerization of the glycosylated product. In order to overcome this issue, we prepared an alternative acceptor for the glycosylation reaction (Scheme 2).

**Scheme 3:**
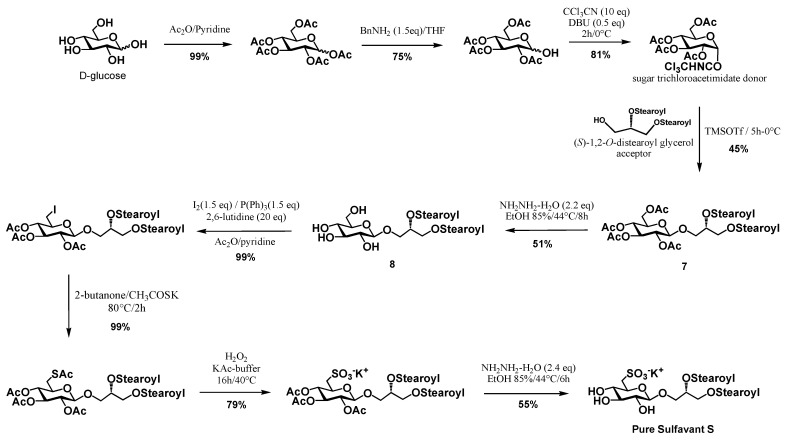
Synthesis of the diastereopure Sulfavant S (**3**).

**Figure 1:**
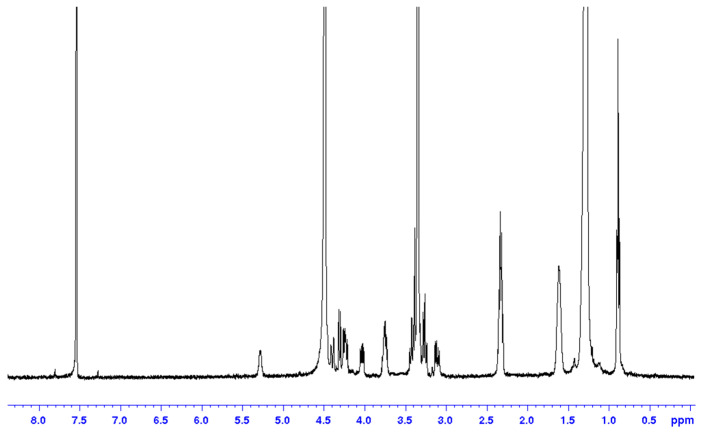
^1^H-NMR of pure **3** in CDCl_3_:CD_3_OD 1:1 at 600MHz.

Starting from the usual *S*-1,2-isopropylideneglycerol, the first step was the protection of the primary hydroxy group by benzylation with benzyl bromide and sodium hydride. Treatment with Dowex H^+^ led to the diol derivative (**5**) from which the distearoyl-benzyl intermediate (**6**) was obtained by acylation with stearic acid and *N,N’*-dicyclohexylcarbodiimide. Without affecting the chirality of the stereocenter at C-2 of glycerol, debenzylation by hydrogenolysis on palladium gave the (*S*)-1,2-*O*-distearoylglycerol acceptor with 35% overall yield. The new acceptor was immediately coupled with the peracetylated glucosyl-trichloroacetimidate donor without loss of stereospecificity. As shown in Scheme 3, after deacetylation of the sugar moiety by hydrazinolysis, the synthesis of the sulfolipid followed the same sequence of reactions previously reported to give pure Sulfavant S [12] (Figure 1).

**Figure 2:**
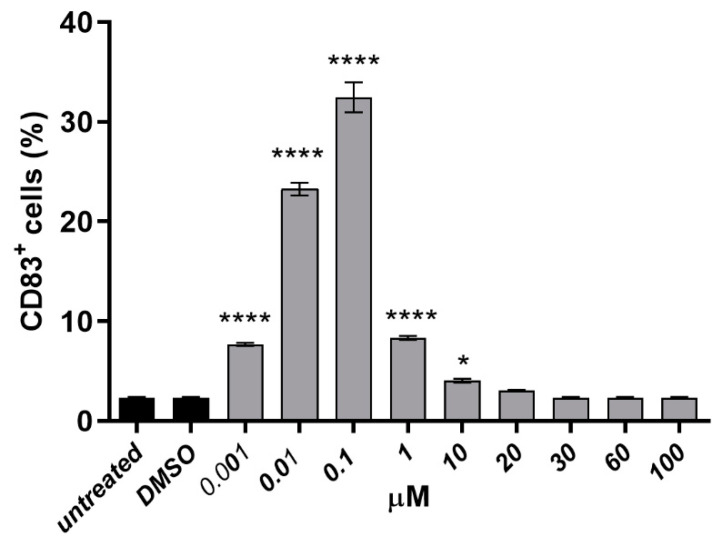
Percentage of CD83^+^ cells stimulated by pure Sulfavant S (**3**); asterisks indicate significant differences from untreated cells; * *p* < 0.5, **** *p* < 0.0001.

Enantiopure Sulfavant S (**3**) was tested for the activation of hDCs derived from blood monocytes [13]. DCs are the most efficient antigen-presenting cells (APCs) [16,17,18,19,20,21,22], often called “nature’s adjuvant” [23] for their ability to induce activation and specific expansion of CD4^+^ helper T (Th) and CD8^+^ cytotoxic T (CTL) lymphocytes. The search for substances able to activate and prepare DCs to face pathogens represents a key tool in the development of new molecular adjuvants for vaccines against tumors or infections [24,25,26]. The effect of compound **3** on hDC maturation was measured by upregulation of CD83, an integral membrane protein belonging to the immunoglobulin superfamily and selectively expressed on mature DCs [27]. The activity of pure Sulfavant S (**3**) was similar to that of Sulfavant R (**2**) [12], with maximum CD83 expression at nanomolar concentration (Figure 2). 

In addition, as previously observed for compounds **1** and **2**, Sulfavant S (**3**) gave a bell-shaped dose activity curve that is common to other lipophilic drugs and reflects the formation of aggregates in the aqueous media [28,29,30,31,32]. In confirmation, Dynamic Light Scattering (DLS) measurements showed that pure **3** formed very small colloidal particles with a hydrodynamic radius of about 50 nm. The size of these particles was very similar to those we have previously observed for the epimer Sulfavant R (**2**) [12], thus proving that pure isomers have very similar biological and chemo-physical properties. Notably, both compounds were 1000-fold more active than Sulfavant A (**1**), which is composed of a 1.3:1 mixture of **2** and **3**. 

In order to test the effect of the aggregation on the immunological response and to test our hypothesis on the reduction of the activity due to mixing of the two active epimers, we analyzed the effect of different combinations of Sulfavant R and Sulfavant S on hDC maturation. 

**Figure 3:**
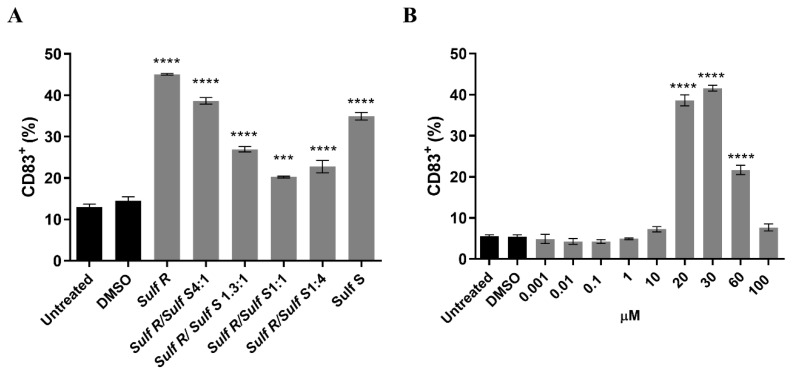
Effect of combination of **2** and **3** on hDC maturation. (**A**) CD83^+^ DC cells triggered by different combinations of **2** and **3** at an overall concentration of 0.1 µM; (**B**) CD83^+^ DC cells triggered by 1.3:1 mixture of **2** and **3** at concentrations from 1 nM to 100 µM; asterisks indicate significant differences from untreated cells; *** *p* < 0.001, **** *p* < 0.0001.

Sulfavant R (**2**) was slightly more active than Sulfavant S (**3**), but their mixtures were always less effective in triggering CD83 expression than the pure molecules (Figure 3A). The addition of the *S* epimer (**3**) to the *R* epimer (**2**) determined a linear decrease of the activity in the range from 100% to 40% of *R.* Further additions revert the response (20% and 0% of *R*) as expected for the formation of mixture where the *S* epimer became progressively predominant. Furthermore, in complete agreement with the response elicited by **1**, the immunomodulatory activity increased to micromolar concentrations by mixture of **2** and **3** with a 1.3:1 ratio, which is identical to the composition of Sulfavant A (**1**) (Figure 3B). On the whole, these experiments proved that the mixing ratio can significantly affect the activity of amphiphilic compounds. In the case of Sulfavants, there is an incredibly large difference between potency of pure products and their mixture, thus determining erroneous evaluation of the therapeutic potential. 

**Figure 4:**
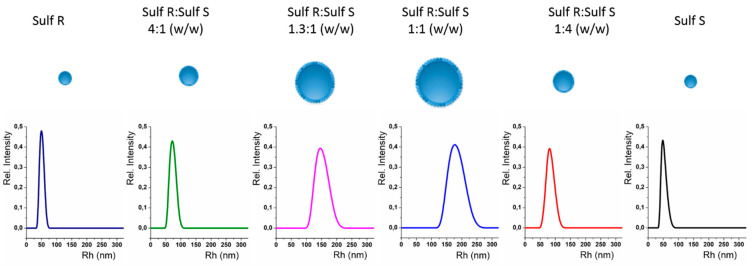
Effect of combination of **2** and **3** on the hydrodynamic radius evaluated by means of Dynamic Light Scattering.

In order to test the role of aggregation on changes of the biological activity of **1–3**, we characterized the suspension of pure products and their mixture in water by Dynamic Light Scattering (DLS) (Figure 4). 

DLS measures showed a constant increase of the aggregate size moving from Sulfavant R (**2**) (about 50 nm) to the 1:1 mixture of Sulfavant R (**2**) and Sulfavant S (**3**) (about 170 nm). In analogy with the effect on the biological activity, further additions of compound **3** shrank the particle up to 50 nm of the pure Sulfavant S (**3**). This surprising behavior was linked to the different way that Sulfavants self-organize in aqueous solution [12]. The evidence that almost identical bioactive molecules (like epimers) could dramatically lose the biological activity as a result of their mixing represents a new paradigm to be evaluated in the study of bioactive lipophilic substances. We have previously suggested that the stability of the final aggregates has a relevant role in influencing the effective bioavailability of free active molecules in equilibrium with self-aggregates [12]. The current results obtained with a mixture of pure Sulfavant R (**2**) and Sulfavant S (**3**) prove, for the first time, that supramolecular organization of mixture of active epimers in aqueous solutions can bias evaluation of their biological and pharmacological potential. 

## 3. Materials and Methods

NMR spectra were recorded on a Bruker Avance-400 (400.13 MHz) and on a Bruker DRX-600 equipped with a TXI CryoProbe in CDCl_3_ and in CDCl_3_:CD_3_OD 1:1 (*δ* values referred to CHCl_3_ and CH_3_OH at 7.26 and 3.34 ppm respectively). HR-MS spectra were acquired by a Q-Exactive Hybrid Quadrupole-Orbitrap mass spectrometer (Thermo Scientific, Waltham, MA, USA). TLC plates (Kieselgel 60 F_254_) and silica gel powder (Kieselgel 60, 0.063–0.200 mm) were from Merck.

All the reagents were purchased from Sigma-Aldrich and used without any further purification. DLS measurements were performed with a homemade instrument composed with a Photocor compact goniometer, a SMD 6000 Laser Quantum 50 mW light source operating at 5325 Å, a photomultiplier (PMT-120-OP/B) and a correlator (Flex02-01D, Correlator.com). 

### 3.1. Dynamic Light Scattering (DLS)

Measurements were performed at (25.00 ± 0.05) °C with temperature controlled through the use of a thermostat bath. In DLS, the intensity autocorrelation function, g^(2)^(t), was measured for the instrument configuration corresponding to the scattering angle of 90°. The intensity autocorrelation function is related to the electric field autocorrelation function through the Siegert relation. The electric field autocorrelation function, g^(1)^(t), is defined as

(1) $$g^{\left(1\right)}(t)=\int_{-\infty }^{+\infty }\tau A(\tau )\exp\left(-\frac{t}{\tau }\right)\;d\;\ln\tau$$

where *τ* = 1/Γ and *q* is the modulus of the scattering vector *q = *4*λn*_0_/*λ sin* (*θ*/2)*,* *n*_0_ = 1.33 is the refractive index of the solvent, *λ* is the incident wavelength and *θ* represents the scattering angle. Evaluation of the relaxation rate Γ distribution allows calculating the translational diffusion coefficient: *D* = Γ/*q*^2^: (J. B. a. R. Pecora, Dynamic Light Scattering: With Applications to Chemistry, Biology, and Physics, Couvire Dover Publications, 2003.)

Inverse Laplace transforms were performed using a variation of the CONTIN algorithm incorporated in Precision Deconvolve software. For spheres diffusing in a continuum medium at infinite dilution, in the approximation of spherical objects, the diffusion coefficient is related to the hydrodynamic radius, *R_h_*, through the Stokes–Einstein equation:

(2) $$R_{h}=\frac{kT}{6\pi \eta_{0}D}$$

where *k* is the Boltzmann constant, *T* is the absolute temperature and *η*_0_ *= 0.89cP* is the solvent viscosity. For not spherical particles, *R_h_* represents the radius of a spherical aggregate with the same diffusion coefficient measured. In the present system, due to the high dilution, it is possible to consider the approximation that *η* ≅ *η*_0_, where *η* represents the solution viscosity. In this hypothesis, Equation (2) can be reasonably used to estimate the averaged hydrodynamic radius of the particles. 

### 3.2. Synthetic Procedures and Characterization of Intermediates 4–7, (S)-1,2-O-Distearoyl Glycerol and Pure Sulfavant S (3)

Compound **4:** Sodium hydride (0.235 g, 0.01 mol) was portion-wise added to (*S*)-(+)-1,2-isopropylideneglycerol (0.6 g, 0.00457 mol) dissolved in THF (7.5 mL), and after 30 min of stirring benzyl bromide (0.85 g, 0.005 mol) was added; after 20 h at 60 °C the mixture was evaporated and purified by silica gel chromatography using a light petroleum ether/diethyl ether gradient to give **4** (1.0 g, 0.0045 mol, 94%); ^1^H-NMR (400 MHz, CDCl_3_): *δ* 7.19–7.08 (5H, overlapped), 4.38 (2H, bs), 4.12 (1H, m), 3.86 (1H, m), 3.58 (1H, m), 3.38 (1H, m), 3.30 (1H, m), 1.27 (3H, s), 1.20 (3H, s); HRESIMS *m/z* 245.1140 [M + Na]^+^ (calcd for C_13_H_18_O_3_Na^+^, 245.1148).

Compound **5:** Compound **4** (1.0 g, 0.0045 mol) was dissolved in methanol/water 95/5 (7 mL) and Dowex H^+^ (7.3 g) was added; after stirring for 1.5 h the mixture was filtered and evaporated giving compound **5** (0.762 g, 0.0042 mol, 93%); ^1^H-NMR (400 MHz, CDCl_3_): *δ* 7.29–7.17 (5H, overlapped, Ph), 4.58 (2H, bs), 3.86 (1H, m), 3.60 (1H, m), 3.52 (1H, m), 3.44 (2H, m); HRESIMS *m/z* 205.0829 [M + Na]^+^ (calcd for C_10_H_14_O_3_Na^+^, 205.0835).

Compound **6:** Compound **5** (0.762 g, 0.0042 mol) was dissolved in anhydrous dichloromethane (7 mL) before addition of 1.1 equiv. of stearic acid, *N,N’*-dicyclohexylcarbodiimide (1.0 g, 0.008 mol) and 4-dimethylaminopyridine (0.51 g, 0.0042 mol) under argon. The reaction mixture was stirred for 16 h at 25 °C; after evaporation under reduced pressure, the mixture was purified by silica gel chromatography using a gradient of petroleum ether/diethyl ether to give compound **6** (2.76 g, 0.0039 mol, 92%) as pale-yellow oil. ^1^H-NMR (400 MHz, CDCl_3_): *δ* 7.29–7.17 (5H, overlapped, Ph), 5.28, (1H, m, H-2), 4.57 (2H, CH_2_Bn), 4.39 (1H, dd, = 3.76 and 11.8 Hz, H-1a), 4.23 (1H, dd, *J* = 6.4 and 11.8 Hz, H-1b), 3.63 (2H, bd, *J* = 5.2 Hz, H-3), 2.33 (4H, m, α-methylene), 1.63 (4H, m, β-methylene), 1.28–1,32 (56H, m, acyl chains CH_2_), 0.83 (6H, overlapped, acyl chains CH_3_); HRESIMS *m/z* 737.6062 [M + Na]^+^ (calcd for C_46_H_82_O_5_Na^+^, 737.6054).

(*S*)-1,2-*O*-distearoyl glycerol**:** Compound **6** (2.76 g, 0.0039 mol) was dissolved in THF/MeOH 1/1 (25 mL) and Pd-C (10%) (0.350 g) was added; after stirring for 16 h at 25 °C the mixture was filtered, evaporated and purified by silica gel chromatography using a light petroleum ether/ethyl acetate gradient to give (*S*)-1,2-*O*-distearoyl glycerol (0.875 g, 0.0014 mol, 35%); ^1^H-NMR (400 MHz, CDCl_3_): *δ* 5.1 (1H, m, H-2), 4.32 (1H, dd, *J* = 4.4 and 11.9 Hz, H-1a), 4.23 (1H, dd, *J* = 5.8 and 11.9 Hz, H-1b), 3.72 (2H, bd, *J* = 4.8 Hz, H-3), 2.33 (4H, m, α-methylene), 1.63 (4H, m, β-methylene), 1.21–1.35 (56H, m, acyl chains CH_2_), 0.85 (6H, overlapped, acyl chains CH_3_); HRESIMS *m/z* 647.5591 [M + Na]^+^ (calcd for C_39_H_76_O_5_Na^+^, 647.5585).

Compound **7:** Peracetylated glucosyl-trichloroacetimidate (0.491 g, 0.001 mol) and (*S*)-1,2-*O*-distearoyl glycerol (0.625 g, 0.001 mol) were dissolved in anhydrous dichloromethane (15 mL), and the solution was kept at 0 °C; then, trimethylsilyl trifluoromethanesulfonate (TMSOTf) (35 µL in 1.5 mL of CH_2_Cl_2_) was added dropwise. The reaction mixture was stirred on activated 3 Å molecular sieves under argon for 5 h at 0 °C and quenched adding triethylamine (150 µL); after evaporation under reduced pressure the crude product was purified by silica gel chromatography using a gradient of petroleum ether/diethyl ether to give compound **7** (0.430 g, 0.00045 mol, 45%). ^1^H-NMR (300 MHz, CDCl_3_): *δ* 5.19 (1H, m), 5.06 (1H, overlapped), 4.99 (1H, t, *J* = 9.5 Hz), 4.88 (1H, dd, *J* = 9.5 and 7.9 Hz), 4.52 (1H, d), 4.02–4.32 (4H, overlapped), 3.93 (1H, dd, *J* = 11.0 and 4.9 Hz), 3.68 (2H, m), 2.3 (4H, m, α-methylenes) 1.98–2.02 (12H, bt), 1.60 (4H, m, β-methylenes), 1.32–1.22 (56H, acyl chains CH_2_), 0.89 (6H, overlapped, acyl chains CH_3_); HRESIMS *m/z* 977.6545 [M + Na]^+^ (calcd for C_53_H_94_O_14_Na, 977.6541).

Sulfavant S **(3**): white solid; ^1^H-NMR (400 MHz, CD_3_OD/CDCl_3_ 1/1): δ values are referred to CHD_2_OD (3.34 ppm and 49.0 ppm): *δ* 5.28 (1H, m, H-2), 4.40 (1H, dd, *J* = 2.7, 12.0 Hz, H-1a), 4.31 (1H, d, *J* = 7.6 H-1′), 4.24 (1H, dd, *J* = 6.9, 12.0 Hz, H-1b), 4.05 (1H, dd, *J* = 5.4, 11.0 Hz, H-3a), 3.79–3.71 (3H, H-3b, H-3′, H-4′), 3.41 (1H, bt, *J* = 8.9 Hz, H-2′), 3.26 (1H, overlapped, H-6′a), 3.25 (1H, overlapped, H-5′), 3.09 (1H, dd, *J* = 7.2, 15.7 Hz, H-6′b), 2.36–2.27 (4H, α-methylenes of stearoyl portions), 1.65–1.56 (4H, β-methylenes of stearoyl portions), 1.36–1.20 (60H, aliphatic methylenes), 0.89 (6H, bt, *J* = 6.0 Hz, 2CH_3_); ^13^C-NMR (100MHz, CD_3_OD:CDCl_3_ 1:1): δ 174.1, 173.7 (C, acyl esters of stearoyl part), 103.2 (CH, C1′), 76.1 (CH, C2′), 73.8 (CH, C5′), 72.4 (CH, C3′), 72.3 (CH, C4′), 70.2 (CH, C2), 68.2 (CH_2_, C3), 63.2 (CH_2_, C1), 53.6 (CH_2_, C6′), 34.2 (CH_2_, α-methylene of stearoyl portion), 32.2–29.0 (CH_2_, methylenes of stearoyl portion), 24.9 (CH_2_, β-methylene of stearoyl portion), 13.8 (CH_3_, methyls of stearoyl portion); HRESIMS *m/z* 849.5772 [M-K]^-^ (calcd for C_45_H_85_O_12_S^-^, 849.5767).

### 3.3. Human Monocyte-Dendritic Cell Differentation

For each assay, human peripheral blood mononuclear cells were isolated from two healthy donors by standard Ficoll density gradient centrifugation. Monocytes were purified from human peripheral blood mononuclear cells using MACS CD14 microbeads (Miltenyi Biotech, Auburn, AL, USA) according to the manufacturer’s recommendation. Purity was checked by staining with a FITC-conjugated anti-CD14 antibody (Milteny Biotech) and FACS analysis and was routinely found to be greater than 98%. Immature DCs were obtained by incubating monocytes at 1 × 10^6^ cells mL^−1^ in an RPMI 1640 medium supplemented with 10% fetal calf serum, 1% L-glutamine 2mM, 1% penicillin and streptomycin, human IL-4 (5 ng/mL), and human GM-CSF (100 ng/mL) for five days.

### 3.4. Cells Staining and Stimulation

After five days in culture, surface staining was performed on monocyte-derived dendritic cells (moDCs) for flow cytometry analysis. moDCs (0.8 × 10^6^ cell/well) were then incubated with synthetic compounds in 12 wells at the reported concentrations. Stimulation with PAM2CSK4 1 µg mL^−1^ (Invivogen) was used as positive control. After 24 h, expression of all surface markers was estimated by using the following conjugated mAbs from Miltenyi Biotec: HLA-DR FITC, CD83 PE and CD86 APC, and analyzed by a flow cytometer (BD ACCURI, BD Bioscience, Milano, Italy) according to standard protocol.

### 3.5. Statistical Analysis

All data were analyzed by one-way ANOVA, followed by the Tukey test for multiple comparisons. A *p*-value less than 0.05 was considered statistically significant. All analyses were performed using the GraphPad Prism 8.00 for Windows software (GraphPad Software, San Diego, CA, USA).

### 3.6. Characterization of Colloid Nanoparticles 

After purification by HPLC, samples were prepared in 1 mL of Millipore water at 0.1 mM of each compound **1**–**3** and mixtures **2** and **3**. After sonication for 30 min at 35 °C, the solutions were maintained at room temperature (20 °C) for 24 h. The mean diffusion coefficients were obtained as an average of at least three measurements at 25 °C.

## 4. Conclusions

Sulfavants are bioactive molecules able to induce DC maturation and trigger an adaptive immune response in vivo [13,14,15]. The activity of these products is largely dependent on the formation of colloidal particles under aqueous conditions [12]. Mixing diastereoisomers **2** and **3**, both active at nanomolar concentrations in pure form, determined a consistent decrease of the biological response in agreement with formation of supramolecular aggregates of different size and stability. Multiple equilibria between free active monomers and different aggregates control the concentration of the products able to bind the cellular targets, thus affecting the results of biological tests and accuracy of the evaluation of the therapeutic potential. In our opinion, this behavior is common to many other lipophilic or amphiphilic compounds, such as natural products, lipopeptides and glycolipids, whose activity can be significantly altered by supramolecular self-assembly in aqueous media. This effect could be more relevant in in vitro assays than in vivo trials since the presence of proteins and other molecules in the body fluids can reduce the tendency of these compounds to aggregate. Sulfavant S (**3**) was synthesized by a modified protocol involving the preparation of (*S*)-1,2-*O*-distearoylglycerol as an alternative acceptor of the glucosyl-trichloroacetimidate donor. This approach allows to overcome the technical issues arising by the low stereocontrol of the coupling reaction and represents a starting point for the preparation of other members of this new family of immunomodulatory agents.
